# Systematic Comparisons of Formulations of Linear Oligolysine Peptides with siRNA and Plasmid DNA

**DOI:** 10.1111/cbdd.12709

**Published:** 2016-02-01

**Authors:** Albert Kwok, David McCarthy, Stephen L. Hart, Aristides D. Tagalakis

**Affiliations:** ^1^ Experimental and Personalised Medicine SectionUCL Institute of Child HealthUniversity College London30 Guilford StreetLondonWC1N 1EHUK; ^2^ UCL School of Pharmacy29‐39 Brunswick SquareLondonWC1N 1AXUK; ^3^ Present address: Department of Clinical Biochemistry University of CambridgeBox 289, Addenbrooke's HospitalCambridgeCB2 0QQUK

**Keywords:** biophysical characteristics, DNA delivery, gene therapy, oligolysine peptide, RNA interference, siRNA delivery

## Abstract

The effects of lysine peptide lengths on DNA and siRNA packaging and delivery were studied using four linear oligolysine peptides with 8 (K8), 16 (K16), 24 (K24) and 32 (K32) lysines. Oligolysine peptides with 16 lysines or longer were effective for stable monodisperse particle formation and optimal transfection efficiency with plasmid DNA (pDNA), but K8 formulations were less stable under anionic heparin challenge and consequently displayed poor transfection efficiency. However, here we show that the oligolysines were not able to package siRNA to form stable complexes, and consequently, siRNA transfection was unsuccessful. These results indicate that the physical structure and length of cationic peptides and their charge ratios are critical parameters for stable particle formation with pDNA and siRNA and that without packaging, delivery and transfection cannot be achieved.

Delivery of nucleic acids to cells is a powerful tool for basic biological research as well as offering the potential for therapeutic development. siRNA delivery in particular is of intense, widespread interest because of the high degree of specificity and potency of these molecules in suppressing gene expression 1. Cationic polymers and liposomes for nanoparticle formulation with siRNA have been widely reported for cell delivery, but improved formulations for *in vivo* delivery are required 2. Optimal siRNA formulations should package siRNA efficiently into stable nanoparticles that bind and enter cells, escape degradation in the endosome and release the siRNA into the cytoplasm where the mRNA and silencing machinery are located 3, 4, 5.

Although both are nucleic acids, DNA and siRNA pose very different challenges in the requirements of a polycationic packaging agent. The most evident is the much larger size of pDNA, and the other is the subcellular site required for activity with plasmids required in the nucleus. Plasmids may be in the range 4–10 kb of double‐stranded DNA, while siRNAs are typically 21–23 nucleotides of double‐stranded RNA so even a single plasmid may provide a focus for aggregation, while many copies of siRNA may be required for the assembly of stable structures and may not be able to form self‐assembling monodisperse complexes. A cationic charge and a small size, 50–200 nm, are crucial for cellular uptake 6, 7, 8. Once internalized within the cells, the nucleic acids need to be released at their appropriate subcellular compartments, with pDNA localized to the nucleus for gene expression and siRNA guided to the processing body (P‐body) in the cytoplasm for gene silencing 3, 4, 5.

Oligolysine peptides have been used widely to form complexes with nucleic acids, especially with pDNA 9, 10, 11, 12, 13, 14, 15, 16, 17, 18, 19, 20, 21; however, studies on using oligolysines for siRNA delivery are limited 22. Oligolysines are an attractive carrier for gene delivery due to the ease of producing such peptides as a monodisperse species in high purity and yield by solid‐phase peptide synthesis, which is very difficult to achieve for longer polylysine chains. We and others have therefore developed methods to modify these oligopeptides with different binding ligands and lipid components for an improved functionality such as cell targeting for gene and drug delivery 23, 24, 25. However, at present, the knowledge regarding which oligolysines would be optimal for nucleic acid delivery, especially for siRNA, is limited. Therefore, we are the first to perform a systematic study on the oligolysines (K8, K16, K24 and K32) to identify the optimal length for pDNA and siRNA delivery. We hypothesize that siRNA has different packaging and delivery requirements compared to pDNA because of the structural and chemical differences. Therefore, we used the chemically well‐defined oligolysine peptides as a model system to systemically dissect the differences between siRNA and pDNA packaging and delivery. Understanding the effects of the cationic peptide structure on DNA and siRNA delivery will provide novel insights into the future development and improvement of nucleic acid delivery systems.

## Materials and Methods

### Transfection reagents and cells

Plasmid pCEP4‐Luc, which was used to generate Neuro‐2A cells stably expressing luciferase, consists of pCEP4 plasmid with the luciferase gene from pGL3 (Invitrogen, Paisley, UK) 1. The pCI‐Luc was created by subcloning the luciferase gene into the pCI plasmid (Promega, Southampton, UK). siRNAs targeting firefly luciferase (siLuc) (GAUAUGGGCUGAAUACAA) 26 and EGFP (siEGFP) (GACGUAAACGGCCACAAGUUC) 27 were synthesized from Dharmacon, Inc (Epsom, UK). The linear lysine peptides used included 8 (K8), 16 (K16), 24 (K24) and 32 (K32) lysines in a row, and they were purchased from ImunnoKontact (Abingdon, UK). Neuro‐2A and Neuro‐2A‐luciferase cells were cultured in DMEM supplemented with 10% foetal calf serum, 1% non‐essential amino acids and sodium pyruvate at 37 °C in humidified atmosphere in 5% carbon dioxide.

### Gel retardation assay

pDNA or siRNA (0.2 *μ*g) was used to form complexes with the linear lysine peptides at different N/P ratios. The complexes were mixed with the loading dye (4 *μ*L) following 30 min of complex formation at room temperature. pDNA complexes were loaded onto a 1% agarose gel, and siRNA complexes were loaded onto a 4% agarose gel for electrophoresis.

### PicoGreen fluorescence quenching experiments

PicoGreen reagent (1:150 *w/v*) (Invitrogen) was added to the pDNA or siRNA (0.2 *μ*g) in TE buffer at room temperature for 10 min. The pDNA or siRNA was then mixed with the linear lysine peptides to form complexes in TE buffer at room temperature for 30 min. A fluorescence plate reader, Fluostar Optima (BMG Labtech, Aylesbury, UK), was used to analyse the quenched fluorescence. To perform the heparin‐mediated complex dissociation assays, heparin sulphate (Sigma‐Aldrich, Gillingham, UK) was added to the complexes formulated in the PicoGreen fluorescence quenching experiments in a range of concentrations (0.02, 0.04, 0.08, 0.16, 0.32, 0.65 and 1.3 U/mL). The naked pDNA or siRNA labelled with PicoGreen was used to normalize the PicoGreen signal detected from the complexes, and values are expressed as percentage relative fluorescence units (RFU).

### Particle sizing and zeta potential measurement

pDNA or siRNA (10 *μ*g) was added to the lysine peptides at different N/P ratios at room temperature, and the complexes were transferred to a low‐volume transparent cuvette. The hydrodynamic size was recorded and analysed by dynamic light scattering (Malvern Nano ZS, Malvern, UK) using the Mark‐Houwink parameters, while the zeta potential was measured by laser Doppler anemometry (LDA) using the Smoluchowski model. DTS, version 5.03, which was provided by the manufacturer, was used for data processing.

### Transmission electron microscopy

For the electron microscopy investigations, the nanocomplexes were prepared as described above (at an N/P ratio of 3:1 for pDNA complexes and 4:1 for siRNA complexes) and were applied onto a glow‐discharged 300‐mesh copper grid coated with a Formvar/carbon support film (Agar Scientific, Stansted, Essex, UK). After a few seconds, the grid was dried by blotting with filter paper. The sample was then negatively stained with 1% uranyl acetate for a few seconds, before blotting with filter paper and air‐dried. Imaging was carried out under a Philips CM120 BioTwin Transmission Electron Microscope (FEI, Eindhoven, Netherlands) and operated at an accelerating voltage of 120 KV.

### pDNA and siRNA transfection

For pDNA transfection, 1 × 10^4^ Neuro‐2A cells were seeded in 96‐well plates at each well 24 h prior to transfection. The transfection complexes were formed by mixing the linear lysine peptides with pCI‐Luc (0.25 *μ*g/well) at a range of N/P ratios in OptiMEM (Invitrogen) and incubated for 30 min at room temperature. The transfection procedure was performed for 4 h at 37 °C, and the transfection complexes were then replaced with the full growth medium. The cells were cultured for 24 h before the luciferase expression analysis.

For siRNA (luciferase‐targeting) transfections, 1 × 10^4^ Neuro‐2A‐luciferase‐expressing cells (Neuro‐2A‐Luc) were seeded in 96‐well plates at each well 24 h prior to transfection. To form the siRNA complexes, the linear lysine peptides were added to siLuc or siEGFP (2.4 pmol) at different N/P ratios in OptiMEM and incubated for 30 min. Following the removal of growth medium on the Neuro‐2A‐Luc cells, the complexes were added to the cells and incubated for 4 h at 37 °C and then the medium was replaced by the full growth medium and cultured for 24 h. Lipofectamine 2000 (Invitrogen) was used as a control, and the transfection procedures were performed in accordance with the manufacturer's instructions.

### Luciferase assay

The transfected cells were harvested for luciferase assays to determine the transfection efficiency. The luciferase activity in the cells were measured by a luciferase assay system (Promega) using a Fluostar Optima luminometer (BMG Labtech). To normalize the luciferase signal from the cells, protein content of cell lysates was measured by the Bio‐Rad protein assay reagent (Bio‐Rad, Hemel Hempstead, UK). Luciferase activity was expressed as RLU per milligram of protein (RLU/mg).

### Flow cytometry and confocal microscopy

pCI‐Luc was labelled with a Cy5 tag following the manufacturer's instructions (Mirus, Cambridge, UK). The Cy5‐labelled pDNA (1 *μ*g in 500 *μ*L) was used to transfect 2 × 10^5^ Neuro‐2A cells in a well of a 12‐well plate for 4 h. The cells were then trypsinized and washed gently with PBS three times and labelled with propidium iodide (PI) (Invitrogen). The cells were analysed by the Epics XL flow cytometer (Beckman, High Wycombe, UK). For confocal microscopy, the Neuro‐2A cells transfected with Cy5‐labelled pDNA complexes were trypsinized and washed gently with PBS three times. The cells were transferred to a slide coated with polylysine and incubated for 30 min at 37 °C. Following washing with PBS three times, the cells were fixed with 4% formaldehyde for 20 min at room temperature and permeabilized with 0.5% Triton in PBS for 5 min at room temperature. The cells were then stained with Alexa Fluor 488 phalloidin (Invitrogen) and DAPI (Vector Labs, Peterborough, UK). The slides were visualized using a confocal microscope (Leica‐Microsystems, Wetzlar, Germany).

### Cell proliferation assay

Cell viability was assessed in 96‐well plates using the CellTiter 96 Aqueous One Solution Cell Proliferation Assay (Promega). Neuro‐2A cells were seeded and transfected as above, and then after 24 h, the medium was substituted for a growth medium containing 20 *μ*L of the CellTiter 96 Aqueous One Solution reagent. Finally, after incubation for 2 h, the absorbance at 490 nm was measured on a FLUOstar Optima spectrophotometer (BMG Labtech). Cell viability for each complex was expressed as a percentage of the viability of control cells.

### Statistical analysis

Data presented in this study were analysed using a two‐tailed, unpaired Student's *t*‐test where applicable.

## Results

### Linear lysine peptides packaged pDNA, but not siRNA

To examine and compare the binding capacity of the lysine‐based peptides with pDNA or siRNA, gel retardation and PicoGreen fluorescence quenching assays were performed.

#### Gel retardation assay

**Figure 1:**
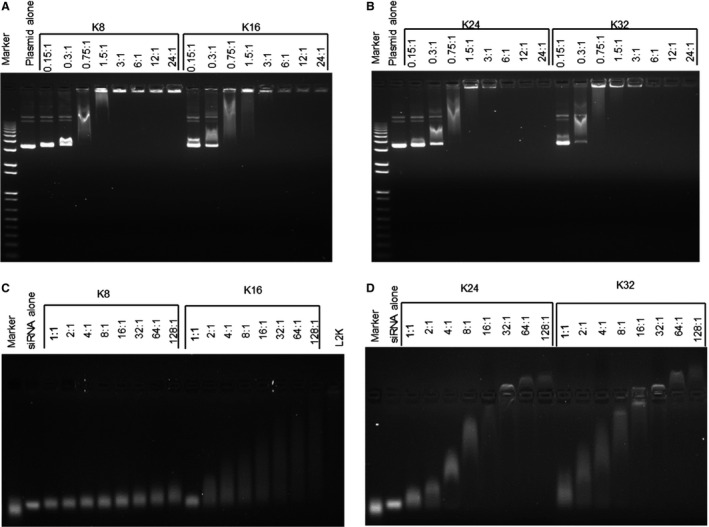
The binding properties of the linear lysine peptides with plasmid DNA or siRNA. The linear lysine peptides (K8, K16, K24 and K32) were mixed with 5 kb pDNA (pCI‐Luc) or siRNA at different N/P ratios for 30 min. The complexes were then run on the 1% agarose gel for the pDNA complexes and 4% agarose gel for the siRNA complexes subsequently. (A) The K8 pDNA and K16 pDNA complexes, (B) the K24 pDNA and K32 pDNA complexes, (C) the K8 siRNA and K16 siRNA complexes and (D) the K24 siRNA and K32 siRNA complexes. The formulations of the complexes are expressed in an N/P ratio.

pDNA complexes were prepared with the four lysine peptides at a range of N/P charge ratios from 0.15 to 24 and analysed for changes in the mobility and ethidium bromide staining intensity of pDNA by agarose gel electrophoresis. All the linear lysine peptides (K8, K16, K24 and K32) bound and retarded pDNA with complete retardation in the well (Figure 1A,B). K8, K16 and K24 completely retarded the pDNA migration at the same N/P ratio of 3:1, while K32 achieved the same retardation at an N/P ratio of 1.5:1. A further increase of the N/P ratio for peptide/pDNA complexes with K16, K24 and K32, but not K8, reduced the ethidium bromide fluorescence intensity in the well of the agarose gel, suggesting fluorescence quenching by further pDNA association. This experiment suggested that K16 was the minimum oligolysine length for optimal pDNA packaging.

A more variable effect of oligolysine length and charge ratio on gel retardation of siRNA was observed (Figure 1C,D). K8 provided a minimal retardation of siRNA compared to the siRNA control even at an N/P ratio of 128:1 (Figure 1C). K16 provided increasing degrees of retardation at increasing charge ratios as evidenced by a smear of increasing length, indicating siRNA complexes of higher molecular weight and/or lower negative charge due to increased peptide association (Figure 1C). K16 could not completely retard siRNA migration even up to an N/P ratio of 128:1, suggesting that siRNA cannot be fully packaged by these peptides. Retardation of siRNA by K24 and K32 peptides increased up to N/P ratios of 16:1 with more compact smearing patterns than those observed with K16, and the complexes were completely retarded in the wells at 16:1 (Figure 1D). Interestingly, at higher charge ratios of the K24 and K32 siRNA complexes (N/P ratios of 32, 64 and 128), siRNA migrated towards the cathode indicating the formation of cationic complexes.

#### PicoGreen fluorescent quenching assay

The binding of lysine peptides to pDNA or siRNA can be assessed by the PicoGreen fluorescent quenching assay. In this assay, pDNA or siRNA were labelled with the PicoGreen fluorescent dye and then mixed with the lysine peptides. Quenching of the fluorescence indicates packaging of the nucleic acid. By comparing the remaining fluorescence of oligolysine complexes with pDNA or siRNA with the fluorescence of naked nucleic acid, the percentage of binding of the pDNA/siRNA by the lysine peptides can be calculated.

**Figure 2:**
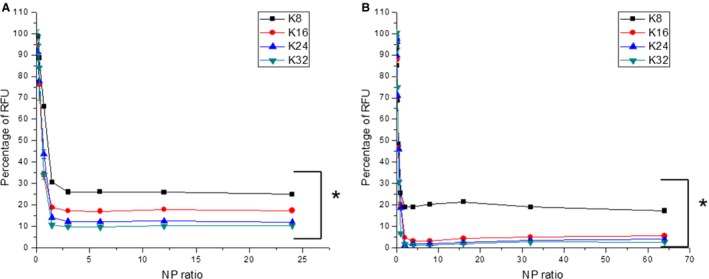
The binding of the linear lysine peptides to (A) pDNA and (B) siRNA. The linear lysine peptides were mixed with PicoGreen‐labelled pDNA or siRNA at different N/P ratios for 30 min. The fluorescence intensity of the complexes was then measured and normalized with the naked pDNA or siRNA. The formulations of the complexes are expressed as an N/P ratio. * denotes a significant difference between the relative fluorescence unit (RFU) of the K8 pDNA/siRNA complexes and the rest of the complexes (p < 0.05).

With pDNA, the lysine peptides achieved maximal quenching of the PicoGreen signal at an N/P ratio of 3 (Figure 2A) with K8, K16, K24 and K32 quenching the fluorescence down to 26%, 17%, 12% and 10% of naked pDNA, respectively (see Figure S1A for the quenching signal at lower N/P ratios). For siRNA, the lysine peptide siRNA complexes all achieved their maximal quenching of the PicoGreen fluorescence at the N/P ratio of 3 (Figure 2B). K8 quenched the PicoGreen signal to 19% of the control, while K16, K24 and K32 decreased the RFU to 5%, 3% and 2% of the naked control, respectively (See Figure S1B for the quenching signal at lower N/P ratios). There is a discrepancy between the siRNA packaging data from the gel retardation assay and the PicoGreen fluorescence quenching assay. During the gel retardation assay, an electric field was applied across the siRNA complexes. As a result, the electrophoretic forces would influence the migration patterns of the complexes dependent on the overall charge of the complexes and the binding strength between cationic peptides and siRNA (i.e. the siRNA migrated to the anode, while the peptides migrated to the cathode). The smear patterns in the gel indicated that either (i) the binding between the peptides and siRNA was too weak and therefore the components of the complexes were being pulled apart by the electrophoretic forces or (ii) the siRNA complexes were heterogeneous in terms of size and charge. However, such an external ‘pulling’ force was not present in the PicoGreen fluorescence quenching assay and this might explain this discrepancy.

### The dissociation properties of the pDNA or siRNA oligolysine complexes

**Figure 3:**
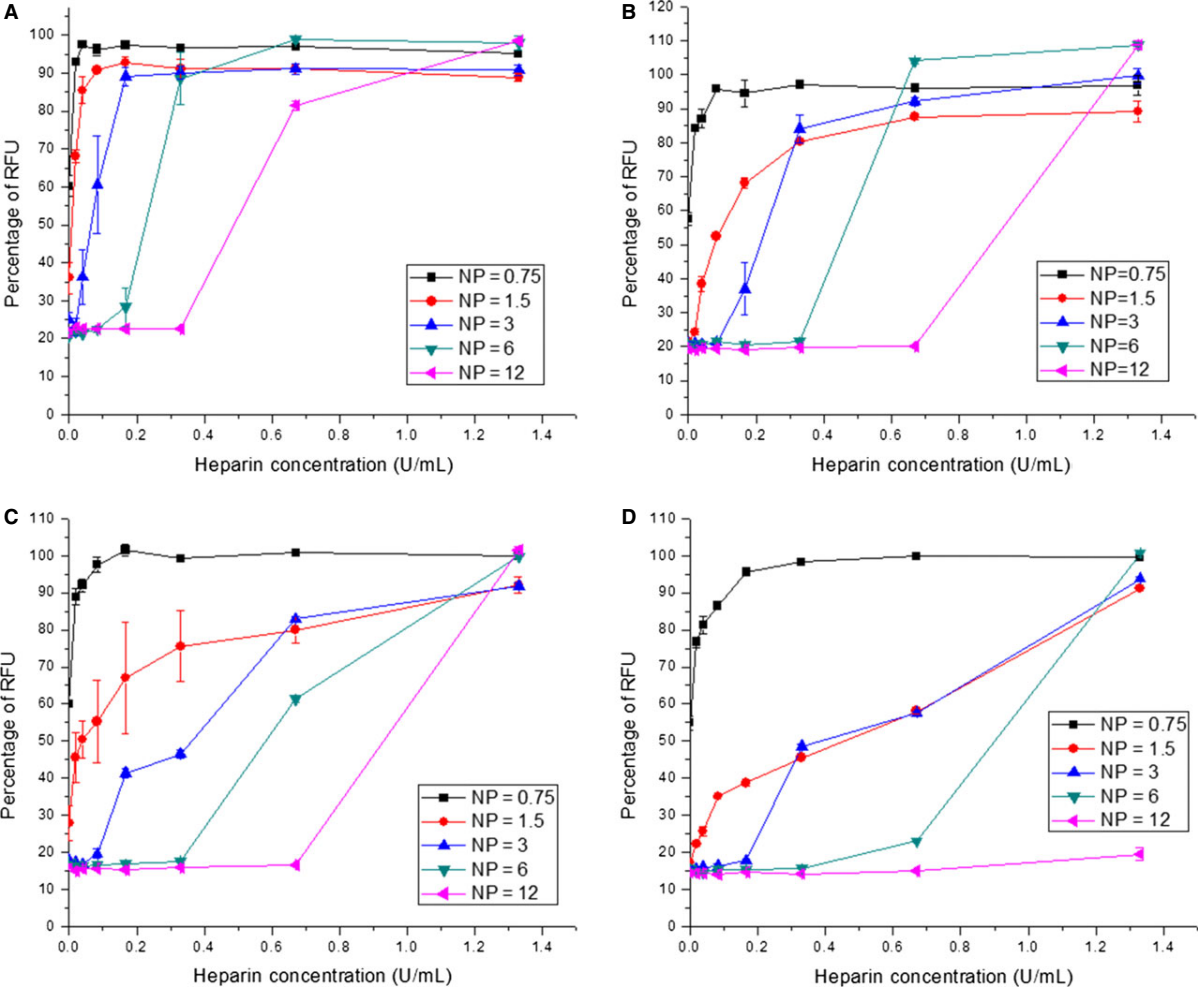
The dissociation properties of linear lysine pDNA complexes. K8, K16, K24 and K32 were mixed with PicoGreen‐labelled pDNA at different N/P ratios for 30 min. Different concentrations of heparin were added to the complexes, and the fluorescence intensity of the complexes was measured. (A) The K8 pDNA complexes, (B) the K16 pDNA complexes, (C) the K24 pDNA complexes and (D) the K32 pDNA complexes. The formulations of the complexes are expressed as an N/P ratio.

The stability and dissociation properties of the pDNA/oligolysine and the siRNA/oligolysine complexes were investigated by an exposure to a range of concentrations of heparin sulphate (0.02–1.3 U/mL) (Figure 3). Heparin is anionic and competes with the nucleic acid binding to the cationic linear lysines, leading to dissociation 28. PicoGreen‐labelled pDNA complexes were prepared with oligolysine peptides at N/P ratios of 0.75:1, 1.5:1, 3:1, 6:1 and 12:1 with pDNA and PicoGreen‐labelled siRNA complexes were prepared at N/P ratios of 1:1, 2:1, 4:1 and 8:1. Complexes were compared for the concentration of heparin required to restore fluorescence RFU values to 50% of the naked nucleic acid, representing a 50% dissociation.

#### Dissociation of the oligolysine pDNA complexes

An increase in the peptide length from K8 to K32 increased the stability of pDNA complexes to dissociation by heparin (Figure 3; Figure S2; Table 1). For K8 pDNA complexes at an N/P ratio of 3, 0.05 U/mL of heparin was required to dissociate complexes by 50% (Figure 3A), while the K16 pDNA complexes required fourfold more heparin (0.2 U/mL) (Figure 3B) and K24 and K32 pDNA complexes required 0.35 and 0.4 U/mL of heparin, respectively (Figure 3C,D). Overall, increasing the size of the linear lysine peptides enhanced the stability of the peptide/pDNA complexes to dissociation by heparin, indicating better binding.

**Table 1:** The dissociation of the peptide/pDNA complexes under the challenge of heparin

N/P ratio	K8			K16			K24			K32		
	Begin to dissociate	50% dissociation	>90% dissociation	Begin to dissociate	50% dissociation	>90% dissociation	Begin to dissociate	50% dissociation	>90% dissociation	Begin to dissociate	50% dissociation	>90% dissociation
0.75	Trace amount	Trace amount	0.02	Trace amount	Trace amount	0.05	Trace amount	Trace amount	0.02	Trace amount	Trace amount	0.1
1.5	Trace amount	Trace amount	0.08	Trace amount	0.05	0.67	Trace amount	0.04	1.1	Trace amount	0.4	1.2
3	Trace amount	0.05	0.17	0.08	0.2	0.55	0.08	0.35	1.1	0.167	0.4	1.2
6	0.8	0.25	0.4	0.33	0.45	0.6	0.33	0.55	1.1	0.33	0.9	1.2

The heparin concentration is indicated in U/mL. Trace amount refers to heparin concentrations lower than 0.02 U/mL.

At N/P ratios above 3, the dissociation of the oligolysine/pDNA complexes required higher amounts of heparin, reflecting their greater stability (Figure 3; Table 1). For example, only a trace amount of heparin was needed to dissociate 50% of the K8 pDNA complexes at an N/P ratio of 1.5, while approximately 0.05, 0.25 and 0.5 U/mL of heparin were required to achieve 50% dissociation of the complexes with N/P ratios of 3, 6 and 12, respectively. A similar relationship between complex stability to heparin and N/P ratios was observed for the K16, K24 and K32 pDNA complexes (Figure 3; Table 1). Interestingly, the K32 pDNA complexes at an N/P ratio of 12 were not dissociated with heparin even at 1.3 U/mL, implying the tight binding between the peptide and the pDNA.

#### The dissociation properties of the siRNA from the linear lysines

**Figure 4:**
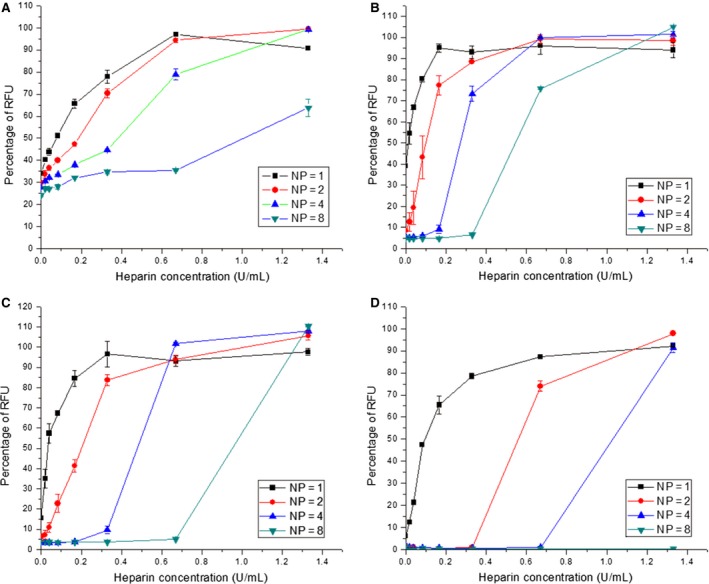
The dissociation properties of linear lysine siRNA complexes. K8, K16, K24 and K32 were mixed with PicoGreen‐labelled siRNA at different N/P ratios for 30 min. Different concentrations of heparin were added to the complexes, and the fluorescence intensity of the complexes was measured. (A) The K8 siRNA complexes, (B) the K16 siRNA complexes, (C) the K24 siRNA complexes and (D) the K32 siRNA complexes. The formulations of the complexes are expressed as an N/P ratio.

Increasing the oligolysine peptide lengths from K16 to K32 at the same N/P ratios also improved siRNA stability (Figure 4; Figure S3; Table 2). For example, 0.1 U/mL of heparin was needed to dissociate 50% of the K16 siRNA complexes at an N/P ratio of 2 (Figure 4B), while twofold more heparin (0.2 U/mL) and 5.5‐fold more heparin (0.55 U/mL) were required to dissociate 50% of the K24 siRNA and K32 siRNA complexes, respectively, at the same N/P ratio (Figure 4C,D). The K8 siRNA complexes, although less quenched, were more resistant to heparin dissociation compared to the K16 siRNA complexes, requiring 0.2 U/mL of heparin at an N/P ratio of 2 for a 50% dissociation (Figure 4A), the same as for K24. The less quenched K8 may suggest that it has a lower dissociation constant (Kd) to anionic molecules such as siRNA. This lower Kd could be due to the fact that K8 has a lower number of cations *per se*, which would be consistent to a previous observation demonstrating that lysine monomers have a lower binding affinity to pDNA compared to polylysines 29. When heparin was added, it competed against the siRNA for the binding to the peptides. In the case of K16, K24 and K32, they had a stronger affinity to the heparin under an increased concentration, and hence, the end result was that the heparin outcompeted the siRNA in the binding. However, the lower Kd of the K8 peptide means that it did not bind strongly to neither the siRNA nor heparin. As a result, K8 would constantly bind and unbind to siRNA/heparin.

**Table 2:** The dissociation of the peptide/siRNA complexes under the challenge of heparin

N/P ratio	K8			K16			K24			K32		
	Begin to dissociate	50% dissociation	>90% dissociation	Begin to dissociate	50% dissociation	>90% dissociation	Begin to dissociate	50% dissociation	>90% dissociation	Begin to dissociate	50% dissociation	>90% dissociation
1	Trace amount	0.07	0.55	Trace amount	Trace amount	0.15	Trace amount	0.05	0.28	Trace amount	0.1	1.1
2	Trace amount	0.2	0.6	Trace amount	0.1	0.35	Trace amount	0.2	0.58	0.33	0.55	1.1
4	Trace amount	0.35	1.1	0.08	0.3	0.55	0.167	0.48	0.65	0.67	1.02	1.3
8	Trace amount	1.02	N/A	0.33	0.55	0.95	0.67	0.95	1.2	N/A	N/A	N/A

The heparin concentration is indicated in U/mL. Trace amount refers to heparin concentrations lower than 0.02 U/mL. N/A means data not available.

Increasing the N/P ratios from 1 to 8 also increased the resistance of siRNA complexes with all oligolysine peptides to heparin dissociation. For instance, the K8 siRNA complexes at N/P ratios of 1, 2, 4 and 8 required 0.07, 0.2, 0.35 and 1.02 U/mL heparin to achieve 50% complex dissociation (Figure 4A), while for K16 siRNA complexes at N/P ratios of 2, 4 and 8, heparin concentrations of 0.1, 0.3 and 0.55 U/mL were needed, respectively (Figure 4B). For K24 siRNA complexes at N/P ratios of 1, 2, 4 and 8, heparin concentrations of 0.05, 0.2, 0.48 and 0.95 U/mL were required for 50% dissociation, respectively (Figure 4C). For K32 siRNA complexes with N/P ratios of 1, 2 and 4, 50% dissociation was achieved with heparin concentrations of 0.1, 0.55 and 1.02 U/mL, respectively (Figure 4D). Furthermore, as observed with the K32/pDNA complexes, no dissociation was observed at the highest N/P ratio of the K32/siRNA complexes.

### Size and zeta potential of the complexes

The average hydrodynamic size of the complexes was determined by dynamic light scattering at a range of charge ratios for each oligolysine peptide (K8, K16, K24 and K32) formulated with pDNA (Table 3). Generally, there was a substantial decrease in size as N/P ratios were increased from 0.75 to 6. For instance, the average size of the K16 pDNA complexes decreased from 89 nm to 46 nm. A similar trend was observed for pDNA complexes formed with K8, K24 and K32 (Table 3).

**Table 3:** The average hydrodynamic size and zeta potential of the linear lysine pDNA complexes

N/P ratios	Average diameter (nm)				Zeta potential (mV)			
	K8	K16	K24	K32	K8	K16	K24	K32
0.75	98 ± 3.8	89.3 ± 1.6	79.5 ± 1.6	156.9 ± 4.1	−17.4 ± 1	−13.3 ± 1	−10.4 ± 1	23 ± 0.6
1.5	66 ± 1.1	64.8 ± 1.2	68.4 ± 1.2	87.4 ± 6.3	27.1 ± 0.9	33.5 ± 1	29.1 ± 0.9	34.5 ± 1 .2
3	55.4 ± 1.0	55.4 ± 0.4	63.6 ± 1	72.4 ± 4.3	34.3 ± 0.7	38.6 ± 2.3	38.9 ± 0.6	11.6 ± 2.5
6	47.9 ± 0.7	46.5 ± 0.9	53.7 ± 0.9	65.4 ± 1.8	32.4 ± 2.7	41.6 ± 1.6	47.1 ± 2.2	52.6 ± 4

In terms of the lysine length and the size of the pDNA complexes, the K8 and K16 pDNA complexes were of similar sizes for N/P ratios of 1.5, 3 and 6. The K24 pDNA complexes were larger than the K8 and K16 pDNA complexes (for N/P ratios of 1.5, 3 and 6), while the K32 pDNA complexes were the largest pDNA complexes for all N/P ratios. This suggested that longer lysine peptides (i.e. longer than K16) would form larger pDNA complexes. The smallest particles were formed by K8 and K16 oligolysine peptides with pDNA at N/P ratios of 6.

The average zeta potential of the linear lysine peptide pDNA complexes became increasingly positive with increases in the N/P ratio from 0.75 to 6 (Table 3). For instance, increasing the N/P ratio of the K8 pDNA complexes from 0.75 to 6 increased the average zeta potential of the complexes from −13 mV to +42 mV. A similar relationship between the N/P ratios and the zeta potential was observed for the other pDNA complexes with K16, K24 and K32. It is worth noting that the K8, K16 and K24 pDNA complexes had positive zeta potential values although they did not seem to be completely retarded in the gel retardation assay. This is because the voltage involved in the zeta potential measurement was less than the voltage used in the gel retardation assay. As a result, there was less ‘pulling’ force exerted in the complexes in the zeta potential measurement, and hence, the relatively weak complexes could remain intact.

A general correlation was observed between the length of the lysine peptides and the zeta potential. Longer oligolysine peptides produced complexes with more positive zeta potentials. For instance, at N/P ratios of 0.75, 3 and 6, the K32 pDNA complexes (e.g. +52.6 mV at an N/P ratio of 6) were the most positive particles, followed by the K24 (e.g. +47.1 mV at an N/P ratio of 6), K16 (e.g. +41.6 mV at an N/P ratio of 6) and K8 pDNA complexes (e.g. +32.4 mV at an N/P ratio of 6), in a descending order. In addition, the K32 pDNA complexes were cationic at all N/P ratios tested.

Generally, for each peptide, higher zeta potential values correlated with smaller particle sizes. However, when comparing the peptides between them, this was not the case. For example, the highest zeta potential value of +53 mV was observed for K32 pDNA complexes at an N/P ratio of 6, but their size of 65 nm was significantly greater than that of K16 pDNA complexes at the same N/P ratio, which had a zeta potential of only +42 mV, but a size of 46 nm. This suggests that other factors, in addition to charge, possibly steric interactions, affected the particle size.

The mean hydrodynamic sizes and zeta potential of complexes formed from the oligolysine peptides and siRNA could not be determined due to the highly polydisperse nature of the complexes (polydispersity index = 1). This suggested that the linear lysine peptides formed highly irregular complexes or a wide range of different‐sized complexes with siRNA.

### The physical structure of the complexes

**Figure 5:**
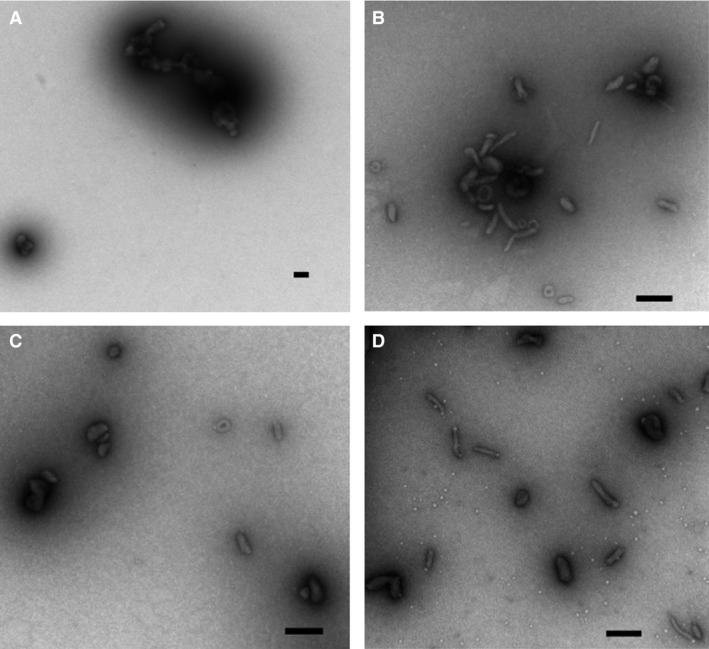
The physical structure of the pDNA complexes. The complexes were prepared at an N/P ratio of 3:1 and were applied onto a glow‐discharged 300‐mesh copper grid coated with a Formvar/carbon support film. The complexes were stained with 1% uranyl acetate before blotting with filter paper and air‐dried. (A) The K8 pDNA complexes, (B) the K16 pDNA complexes, (C) the K24 pDNA complexes and (D) the K32 pDNA complexes. The bar represents 100 nm.

To further characterize the physical structures of the complexes, transmission electron microscopy (TEM) was performed. All the linear lysine peptides used in the study formed complexes with pDNA with a size ranging from 50 to 100 nm (Figure 5A–D). While the K8 pDNA complexes were mainly spherical or rope‐like shaped (length close to 100 nm or longer), we observed toroidal and shorter rod‐shaped (length close to 50 nm) pDNA complexes from K16, K24 and K32 complexes.

**Figure 6:**
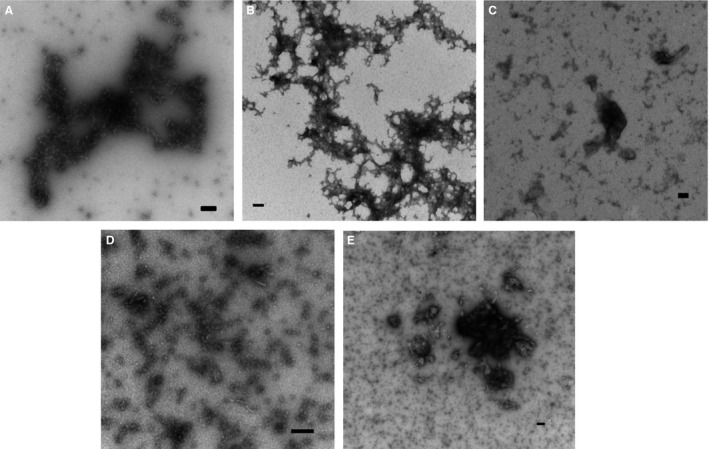
The physical structure of the pDNA complexes. The complexes were prepared at an N/P ratio of 4:1 and were applied onto a glow‐discharged 300‐mesh copper grid coated with a Formvar/carbon support film. The complexes were stained with 1% uranyl acetate before blotting with filter paper and air‐dried. (A) siRNA alone, (B) the K8 siRNA complexes, (C) the K16 siRNA complexes, (D) the K24 siRNA complexes and (E) the K32 siRNA complexes. The bar represents 100 nm.

When investigating the physical structures of the siRNA complexes (Figure 6A–E), we found that all the lysine peptides formed large aggregates with siRNA, instead of forming monodisperse nanoparticles. This observation is consistent with the hydrodynamic size measurements.

### Cell binding and internalization of oligolysine formulations

**Figure 7:**
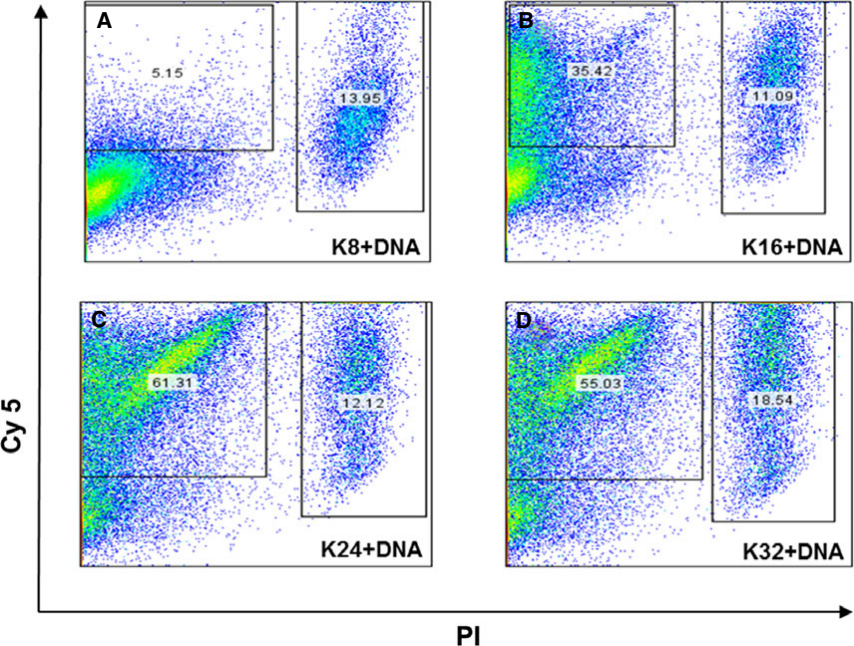
Cellular binding/uptake efficiencies of linear lysine pDNA complexes. Neuro‐2A cells were seeded 24 h before transfection. The complexes were made by mixing the peptides with Cy5‐labelled pCI‐Luc for 30 min. Following the removal of full growth medium, complexes were overlaid to the cells for 4 h. The cells were harvested for analysis after transfection. For flow cytometry analysis, propidium iodide (PI) was used to estimate the cell viability following transfection: (A) cells exposed to K8 pDNA complexes at 3:1 N/P ratio, (B) cells exposed to K16 pDNA complexes at 3:1 N/P ratio, (C) cells exposed to K24 pDNA complexes at 3:1 N/P ratio and (D) cells exposed to K32 pDNA complexes at 3:1 N/P ratio.

Oligolysine pDNA formulations were prepared with pDNA labelled with Cy5 at an N/P ratio of 3 and incubated with Neuro‐2A cells for 4 h and then analysed by flow cytometry (Figure 7A–D). Cells were also stained with PI to distinguish live cells from dead cells. It was found that cell binding of K8 complexes was very poor, with only 5% of live cells associated with Cy5‐labelled complexes. However, complexes containing K16, K24 and K32 peptides were associated with 35%, 61% and 55% of live cells, respectively (Figure 7A–D). Complexes with K32 appeared to be associated with an increased number of dead cells (19%) compared to K8, K16 and K24 formulations with 14%, 11% and 12%, respectively, suggesting that some cytotoxicity was associated with the longest oligolysine peptide.

**Figure 8:**
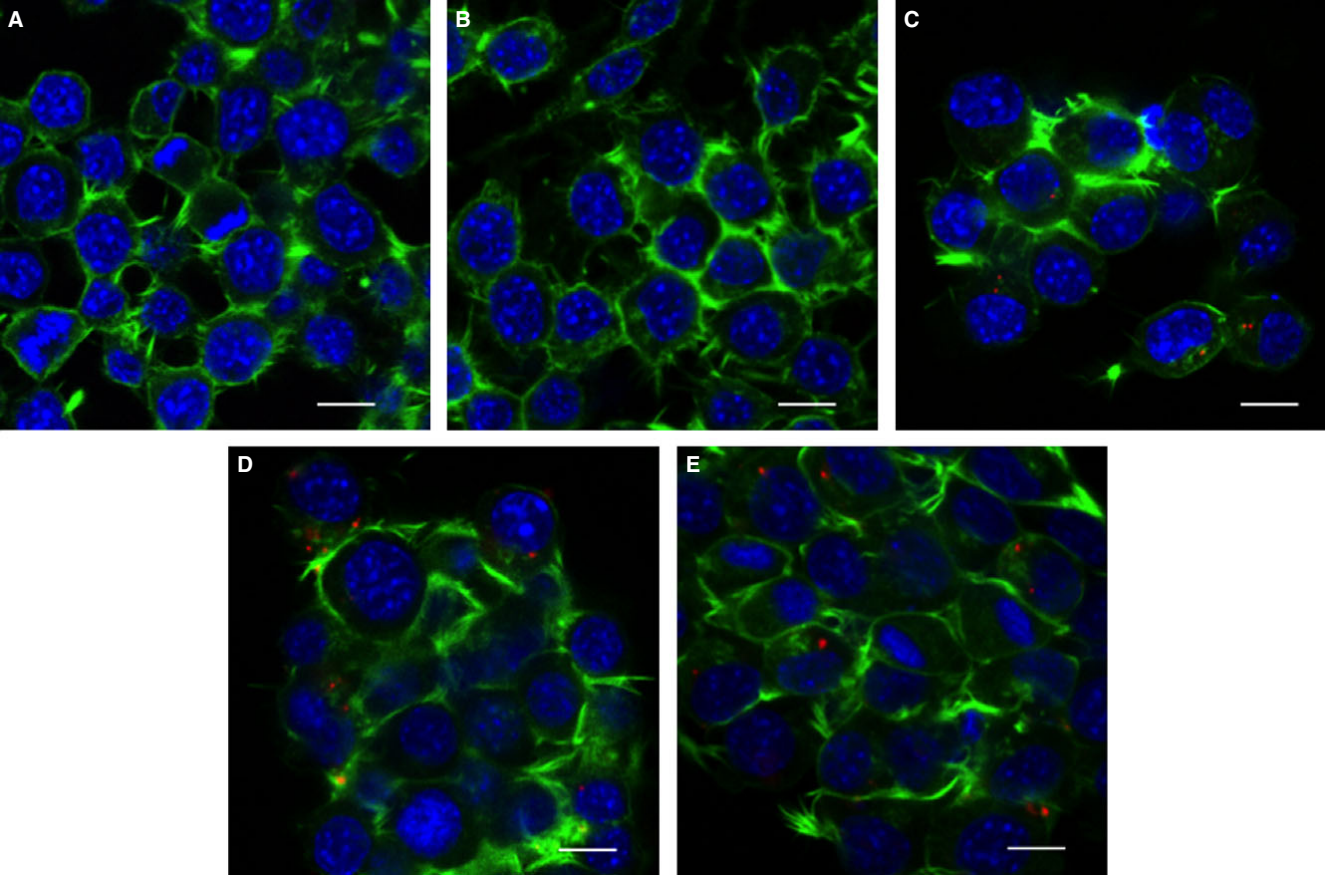
Cellular uptake and localization of linear lysine pDNA complexes. Neuro‐2A cells were transfected at an N/P ratio of 3:1 as described in Figure 7. For confocal microscopy, cells were washed and stained with phalloidin for the F‐actin on the cell membrane (green), DAPI for the nucleus (blue) and Cy5 for the complexes (red). (A) Untreated cells, (B) cells exposed to K8 pDNA complexes, (C) cells exposed to K16 pDNA complexes, (D) cells exposed to K24 pDNA complexes and (E) cells exposed to K32 pDNA complexes. Scale bar = 15 *μ*m.

Cells were transfected with Cy5‐labelled complexes containing the oligolysine peptides (an N/P ratio of 3:1) and analysed by confocal microscopy. Analysis revealed that Cy5‐labelled particles within the cells treated with K16 formulations localized into the cytoplasm and perinuclear regions (Figure 8). Similar patterns were observed with K24 and K32 peptides, but for K8 formulations, no Cy5‐labelled cells were detected. This evidence was consistent with flow cytometry data which showed that K16, K24 and K32 pDNA complexes were capable of entering cells within 4 h of incubation, while K8 peptide pDNA complexes mostly failed to enter cells.

To compare the siRNA delivery efficiency of the oligolysines, the lysine peptides were prepared with siRNA labelled with Cy3 and incubated with Neuro‐2A cells for 4 h and then analysed by flow cytometry. However, we did not observe any oligolysine siRNA complex uptake by the cells (data not shown).

### Transfection studies of the lysine‐based peptide pDNA complexes

**Figure 9:**
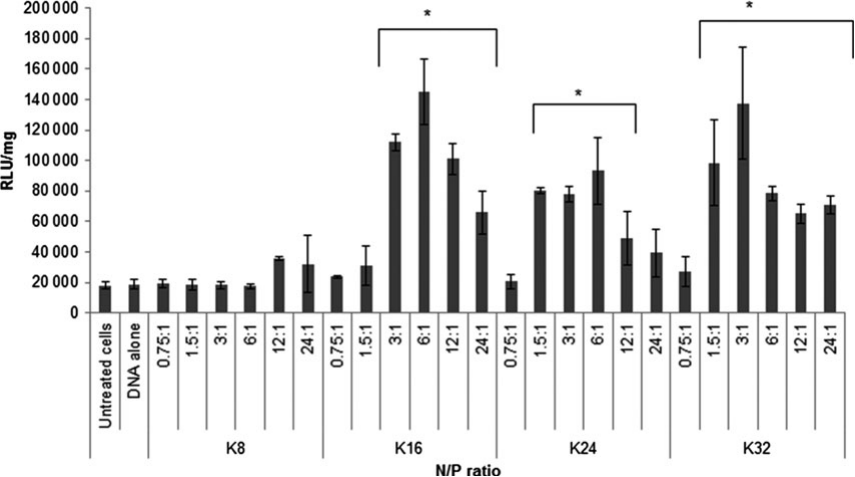
Plasmid transfection efficiency mediated by linear lysine pDNA complexes. Neuro‐2A cells were seeded 24 h before transfection. The complexes were made by mixing peptides (K8, K16, K24 and K32) with pCI‐Luc in different N/P ratios for 30 min. Following removal of the full growth medium, complexes were overlaid to the cells for 4 h. After removing the transfection complexes, the full growth medium was added to the cells. Luciferase expression in the cells was analysed 24 h post‐transfection to estimate the transfection efficiencies of the complexes. The formulations of the complexes are expressed as an N/P ratio. * denotes the significant difference in the RLU/mg between the untreated cells and the transfected cells (p < 0.05).

The K16 pDNA complexes mediated detectable transfection levels at an N/P ratio of 3, with a small increase (1.3‐fold) in transfection efficiency at an N/P ratio of 6 (Figure 9). However, the transfection efficiency decreased when the N/P ratios were further increased to 12 and 24. The K24 pDNA complexes transfected cells starting from an N/P ratio of 1.5 with similarly effective transfection at N/P ratios of 3 and 6. Further increases in the N/P ratio of K24 pDNA complexes decreased their transfection efficiency. The K32 pDNA complexes achieved optimal transfection efficiency at an N/P ratio of 3, and again, increasing the N/P ratio of these complexes decreased the transfection efficiency. K8 pDNA complexes only achieved a detectable transgene expression at an N/P ratio of 12:1. In summary, K16 at an N/P ratio of 3 was effective for pDNA delivery; however, increasing the length to K24 and K32 did not further improve the transfection efficiency.

**Figure 10:**
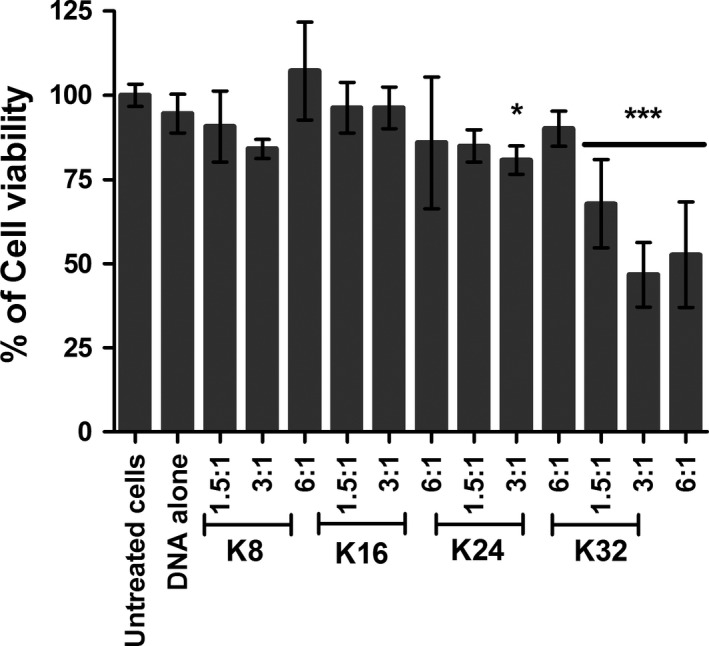
Cytotoxicity induced by transfection of linear lysine pDNA complexes. Neuro‐2A cells were seeded 24 h before transfection. The complexes were made by mixing peptides (K8, K16, K24 and K32) with pCI‐Luc in different N/P ratios for 30 min. Following removal of the full growth medium, complexes were overlaid to the cells for 4 h. After removing the transfection complexes, the full growth medium was added to the cells. Cell viability was analysed 24 h post‐transfection to estimate the cytotoxicity caused by the transfection. The formulations of the complexes are expressed as an N/P ratio. * and *** denote the significant difference in the cell viability between the untreated cells and the transfected cells at p < 0.05 and p < 0.001, respectively.

Cytotoxicity mediated by the transfection using the linear lysine peptides at different N/P ratios was further analysed (Figure 10). There was a trend that the pDNA complexes formed by K8, K16 and K24 would mediate <10% of cell death, while the K32 pDNA complexes were significantly more toxic than other complexes, inducing up to 50% of cell toxicity (N/P ratios of 3:1 and 6:1) 24 h post‐transfection.

### Gene silencing of the lysine‐based peptide siRNA complexes

The results of the siRNA transfection using the oligolysine peptides revealed no significant luciferase knockdown, whereas commercial Lipofectamine 2000 (L2000) siRNA complexes induced 80% gene knockdown (Figure S4).

## Discussion

Oligolysine peptides have been under investigation as components of vector formulations for the packaging and delivery of both pDNA and siRNA 10, 11, 12, 13, 14, 15, 16, 17, 18, 19, 22, 24, 30, 31, 32, 33. Despite reports showing that these peptides can deliver both kinds of nucleic acid to cells, systematic studies on the relationship between the nucleic acid and peptide structure with complex formation, stability and transfection efficiency have been limited. Such interactions and optimal requirements might be expected to differ considerably owing to the considerable size differences between the two nucleic acids and to their different subcellular sites of activity 1. In this study, linear oligolysine peptides with four different lengths (K8, K16, K24 and K32) were systematically compared for their interactions with both siRNA and pDNA. This should allow us to understand the implications of the differences in the physical structures, chemical components and sites of activity in delivering the nucleic acids. Understanding such implications is crucial for further developing optimal peptide‐based nucleic acid delivery systems that could easily be modified to acquire specific functionalities such as cell specific targeting and biocompatibility 2, 12, 13, 30.

An effective pDNA transfection reagent would demonstrate certain biophysical properties when forming complexes with pDNA. Such properties include the ability to package pDNA, unload pDNA and form small, discrete, stable particles. Studies were performed to investigate the ability of each peptide to package the nucleic acids at a range of charge ratios, calculated from the N/P ratios for each formulation. We observed that each peptide formed pDNA complexes at an N/P ratio of 3, despite the fact that longer peptides bound to pDNA with a greater affinity (e.g. in the PicoGreen fluorescence assay, K24 and K32 achieved similarly high levels of quenching (~90%) than K16 (~85%) or K8 (~75%)). Such findings were consistent with others 15, 16, 34, 35, 36, although different lysine lengths were used in other studies. Taken together, we identified a trend that the longer the lysine lengths, the more compact the pDNA complexes.

A necessary corollary of efficient pDNA packaging is that for efficient gene expression, the oligolysine peptide must release the pDNA within the cell into the nucleus. To compare particle stability and dissociation potential, oligolysine complexes with pDNA were incubated with anionic heparin, mimicking the intracellular environment where high concentrations of anions may be encountered, such as RNA, DNA, sialic acid 16, 30. Complexes were preformed with PicoGreen‐labelled pDNA to achieve maximal quenching and then incubated with increasing concentrations of heparin, and stability was quantified as the amount of heparin required to restore fluorescence levels to 50% of naked pDNA. The heparin dissociation assay indicated complex stability at the same N/P ratio in the order of K32 > K24 > K16 > K8. For example, at the optimal N/P ratio of 3 for packaging, the concentrations increased from 0.1 U/mL for K8, to 0.2 U/mL for K16 and 0.4 U/mL for both K24 and K32 peptides. Therefore, higher concentrations of heparin were required to dissociate complexes formulated with longer lysines at the same N/P ratios, suggesting that those complexes would be more stable to dissociation in both the extracellular and intracellular environments.

Biophysical analysis of pDNA complexes revealed that at sufficiently high N/P ratios, all oligolysine reagents used in this study formed stable, positively charged pDNA complexes. Similar to the observation from others 15, 37, 38, we found that longer lysine peptides tended to form larger pDNA complexes with sizes ranging from <50 nm for K8 peptides to 72 nm for K32 peptides at an N/P ratio of 3, and a similar pattern at other charge ratios. Larger peptides also formed complexes with higher zeta potential values than those made with shorter peptides ranging from +34 mV for K8 complexes to +42 mV for K32 complexes at the N/P ratio of 3. Thus, longer peptides were forming larger particles with higher zeta potential values. The data from TEM confirmed that all the lysine peptides formed uniform nanoparticles with pDNA. The K8 pDNA complexes were mainly in the less compact spherical and rope‐like (length close to 100 nm or longer) shapes, while the K16, K24 and K32 complexes were in the more compact shorter rod (length close to 50 nm) and toroidal forms. The longer lysine peptides are more effective in condensing pDNA because they can provide more cations for pDNA binding 39, 40.

Interactions of oligolysine peptide/pDNA complexes with cells were then investigated, from cell binding and uptake to transfection efficiencies. The biophysical analysis had suggested that at an N/P ratio of 3, all the complexes were positively charged, which would permit charge‐mediated cell binding, and small enough to be internalized by clathrin‐ or caveolae‐mediated endocytosis, the first steps in the transfection pathway 41, 42, 43. Analysis of cell binding and uptake by flow cytometry for oligolysine complexes containing Cy5‐labelled pDNA revealed that pDNA complexes made with K16, K24 and K32 peptides were efficiently taken up by cells, with K32 complexes displaying more toxicity, while K8 complexes were poorly taken up. The toxicity of the K32 peptide can be explained by the fact that K32 has a strong affinity to pDNA, which may in turn result in excessive binding of genomic pDNA in cells, upsetting the gene regulation and eventually causing cell death. Confocal fluorescence microscopy analysis of K16, K24 and K32 pDNA complexes further revealed that Cy5‐labelled pDNA was detectable within cells after 4 h of incubation at 37 °C, while no plasmid uptake was detectable with K8 pDNA complexes. Cell uptake studies suggested that the low stability of K8 pDNA complexes may have dissociated at the cell surface due to electrostatic forces of anionic surface moieties such as glycoproteins, sialic acid as reported for other poor efficiency formulations 44. Being rope‐like and spherical in shapes, the K8 pDNA complexes are less favourable for endocytosis compared to the more compact toroid and shorter rod pDNA particles 45, 46. Luciferase reporter gene transfection was detected from incubations of pDNA complexes with peptides K16, K24 and K32, at optimal N/P ratios of 3 or 6. K8 complexes, however, displayed very low transfection levels across the full range of charge ratios (0.75–24), consistent with the poor efficiency of binding and uptake 47, 48, 49.

Peptides K8, K16, K24 and K32 all formed small, cationic, stable nanoparticles, but differed in the efficiency of pDNA packaging in proportion to the oligolysine length. The ease of dissociation of the same complexes was inversely proportional to peptide length 34, 35, 36. Overall, in cell transfection studies, peptide K16 appeared to offer the minimum effective oligolysine peptide length in pDNA complexes, with the ideal balance of particle size, stability and charge permitting cell binding, uptake and transfection. However, the biophysical differences between K16, K24 and K32 were quite subtle, which was reflected in their similar transfection efficiencies, while the K8 peptide pDNA complexes appeared not to package as well as the other peptides, or provide stability to heparin challenge, which were the major biophysical correlations with the poor transfection efficiency.

Studies on the biophysical characteristics and delivery of the oligolysine siRNA complexes revealed a different story to the pDNA complexes. PicoGreen fluorescence quenching assays showed that the lysine peptides appeared to associate with the siRNA with maximal fluorescence quenching at N/P ratios around 2–4, as observed with pDNA. However, the extent of packaging with K8 was significantly lower than that for the other three peptides. Gel retardation assays revealed a smear of siRNA complexes formed with K8 and K16 at all N/P ratios tested, whereas peptides K24 and K32 may completely retard the siRNA to the wells at very high N/P ratios between 24 and 32. When the N/P ratio of K24 and K32 was further increased, the complexes migrated to the cathode. These data were consistent to the failed attempts at measuring particle size and charge due to high polydispersity of all complexes. The nature of the high polydispersity of all the complexes was confirmed by the TEM. While the oligolysines could bind to siRNA, the peptides might not be able to form a particle with a positive surface charge. As a result, all the particles formed at the initial stage of complex formation would aggregate under van der Waal's forces 15. Therefore, linear lysine peptides were not able to package the siRNA to form complexes with the optimal size and shape for cellular uptake 45, 46. Unsurprisingly, delivery of luciferase siRNA to Neuro‐2A‐Luc cells failed to induce luciferase silencing. This study shows that linear lysine peptides, without any other components, are efficient and potentially useful reagents for packaging and delivery of pDNA, but not siRNA. This difference may be explained by the relatively small size and greater rigidity of siRNA compared to those of pDNA. siRNA is a double‐stranded nucleic acid with 21–23 base pairs. Compared to the much larger pDNA (usually 1–10 kb), siRNA has limited opportunity for intermolecular electrostatic interactions with cationic oligolysine peptides. Furthermore, the length of siRNA is approximately 6 nm, which is considerably shorter than its 70 nm persistence length (persistence length is the minimum length required for a double‐stranded nucleic acid to fold in any given direction). Therefore, siRNA is a rigid and inflexible structure with minimum rotational freedom 50, 51. However, pDNA (its persistence length = 40–50 nm) is considerably longer than its persistence length and therefore has a structure that allows it to fold more flexibly 52. To package siRNA, a structure with high folding flexibility may be required to encapsulate the small nucleic acid. The linear lysine peptides used in this study are short and of limited folding flexibility; this may explain why they are incapable of condensing siRNA into monodisperse complexes for functionality.

Overall, our results have shown that the generic belief that higher molecular weight cationic polymers would confer a better binding and condensing of nucleic acids for better delivery is incomplete and that we need to consider the physical structure as well as the number of charges on the nucleic acid delivery agent according to the type of nucleic acids to be delivered. In the case of siRNA delivery, a branched structure may be more favourable than a linear structure because a branched structure has higher rotational freedom 2, 19, 53. Indeed, we have observed that a small‐branched lysine peptide is able to package siRNA into monodisperse complexes (data not shown). Previously, we demonstrated that branched PEI is more effective in siRNA packaging and delivery compared to linear PEI 1. Other studies have also shown that larger and branched polymers such as hyperbranched polylysines 54, dendrimers 55, branched PEI 1, 56, histidylated polylysine peptides 57, 58, 59, 60, 61 and chitosan 27, 62, which all are large‐branched structures, are all useful for siRNA delivery. This may indicate that a flexible structure would be more able to package and deliver siRNA. Further studies to elucidate the structure–function relationship of the degree of branched reagents and siRNA or DNA delivery would help improve the current nucleic acid delivery systems.

## Conclusions

The effects of different lysine peptide lengths on pDNA and siRNA packaging and delivery were studied. Four linear oligolysines, K8, K16, K24 and K32, were used. We demonstrated by fluorescence quenching and gel shift assays, dynamic light scattering, laser Doppler anemometry and TEM that the oligolysine peptides formed positively charged monodisperse complexes with pDNA although K8 formulations were less quenched and less resistant to heparin dissociation assays and consequently displayed poor transfection efficiency. In these experiments, the K16 peptides were the minimally effective peptides for stable particle formation and optimal transfection efficiency. For siRNA packaging, evidence from gel shift assays, dynamic light scattering and TEM indicated that all the linear oligolysines tested were not able to package siRNA to form monodisperse complexes, and consequently, siRNA transfection was unsuccessful. The inability of the oligolysines to condense siRNA could be due to the relatively small size and greater rigidity of the siRNA, and therefore, a more flexible structure such as branched polymers or dendrimers could be more useful to condense siRNA. All in all, our results indicate that the length and the physical structure of the peptides and the charge ratio are integral parameters to consider for effective packaging and delivery of nucleic acids such as plasmid DNA and siRNA.

## Conflict of Interest

No conflict of interests to declare.

## Supporting information


**Figure S1.** The binding of the linear lysine peptides to (A) pDNA and (B) siRNA at lower N/P ratios.
**Figure S2.** The dissociation properties of linear lysine pDNA complexes.
**Figure S3.** The dissociation properties of linear lysine siRNA complexes.
**Figure S4.** siRNA transfection efficiency mediated by the linear lysine peptides with siRNA complexes.

## References

[1] Kwok A. , Hart S.L. (2011) Comparative structural and functional studies of nanoparticle formulations for DNA and siRNA delivery. Nanomedicine;7:210–219. 20709624 10.1016/j.nano.2010.07.005

[2] Kwok A. (2013) The challenges and current advances in delivering RNAi as therapeutics In: Barciszewski J., Erdmann V.A., editors. The Challenges and Current Advances in Delivering RNAi as Therapeutics. Berlin: Springer; p. 189–224.

[3] Jagannath A. , Wood M.J. (2009) Localization of double‐stranded small interfering RNA to cytoplasmic processing bodies is Ago2 dependent and results in up‐regulation of GW182 and Argonaute‐2. Mol Biol Cell;20:521–529. 18946079 10.1091/mbc.E08-08-0796 PMC2613116

[4] Sen G.L. , Blau H.M. (2005) Argonaute 2/RISC resides in sites of mammalian mRNA decay known as cytoplasmic bodies. Nat Cell Biol;7:633–636. 15908945 10.1038/ncb1265

[5] Gary D.J. , Puri N. , Won Y.Y. (2007) Polymer‐based siRNA delivery: perspectives on the fundamental and phenomenological distinctions from polymer‐based DNA delivery. J Control Release;121:64–73. 17588702 10.1016/j.jconrel.2007.05.021

[6] Gao H. , Shi W. , Freund L.B. (2005) Mechanics of receptor‐mediated endocytosis. Proc Natl Acad Sci U S A;102:9469–9474. 15972807 10.1073/pnas.0503879102 PMC1172266

[7] Lawrence M.S. , Foellmer H.G. , Elsworth J.D. , Kim J.H. , Leranth C. , Kozlowski D.A. , Bothwell A.L. , Davidson B.L. , Bohn M.C. , Redmond D.E. Jr (1999) Inflammatory responses and their impact on beta‐galactosidase transgene expression following adenovirus vector delivery to the primate caudate nucleus. Gene Ther;6:1368–1379. 10467361 10.1038/sj.gt.3300958

[8] Aoki H. , Satoh M. , Mitsuzuka K. , Ito A. , Saito S. , Funato T. , Endoh M. , Takahashi T. , Arai Y. (2004) Inhibition of motility and invasiveness of renal cell carcinoma induced by short interfering RNA transfection of beta 1,4GalNAc transferase. FEBS Lett;567:203–208. 15178323 10.1016/j.febslet.2004.04.060

[9] Dash P.R. , Read M.L. , Barrett L.B. , Wolfert M.A. , Seymour L.W. (1999) Factors affecting blood clearance and in vivo distribution of polyelectrolyte complexes for gene delivery. Gene Ther;6:643–650. 10476224 10.1038/sj.gt.3300843

[10] Ramsay E. , Hadgraft J. , Birchall J. , Gumbleton M. (2000) Examination of the biophysical interaction between plasmid DNA and the polycations, polylysine and polyornithine, as a basis for their differential gene transfection in‐vitro. Int J Pharm;210:97–107. 11163991 10.1016/s0378-5173(00)00571-8

[11] Wu G.Y. , Wu C.H. (1987) Receptor‐mediated in vitro gene transformation by a soluble DNA carrier system. J Biol Chem;262:4429–4432. 3558345

[12] Harbottle R.P. , Cooper R.G. , Hart S.L. , Ladhoff A. , McKay T. , Knight A.M. , Wagner E. , Miller A.D. , Coutelle C. (1998) An RGD‐oligolysine peptide: a prototype construct for integrin‐mediated gene delivery. Hum Gene Ther;9:1037–1047. 9607415 10.1089/hum.1998.9.7-1037

[13] Tagalakis A.D. , Grosse S.M. , Meng Q.H. , Mustapa M.F. , Kwok A. , Salehi S.E. , Tabor A.B. , Hailes H.C. , Hart S.L. (2011) Integrin‐targeted nanocomplexes for tumour specific delivery and therapy by systemic administration. Biomaterials;32:1370–1376. 21074847 10.1016/j.biomaterials.2010.10.037

[14] Ramsay E. , Gumbleton M. (2002) Polylysine and polyornithine gene transfer complexes: a study of complex stability and cellular uptake as a basis for their differential in‐vitro transfection efficiency. J Drug Target;10:1–9. 11996081 10.1080/10611860290007487

[15] Kwoh D.Y. , Coffin C.C. , Lollo C.P. , Jovenal J. , Banaszczyk M.G. , Mullen P. , Phillips A. , Amini A. , Fabrycki J. , Bartholomew R.M. , Brostoff S.W. , Carlo D.J. (1999) Stabilization of poly‐L‐lysine/DNA polyplexes for in vivo gene delivery to the liver. Biochim Biophys Acta;1444:171–190. 10023051 10.1016/s0167-4781(98)00274-7

[16] Mannisto M. , Reinisalo M. , Ruponen M. , Honkakoski P. , Tammi M. , Urtti A. (2007) Polyplex‐mediated gene transfer and cell cycle: effect of carrier on cellular uptake and intracellular kinetics, and significance of glycosaminoglycans. J Gene Med;9:479–487. 17410614 10.1002/jgm.1035

[17] Ziady A.G. , Ferkol T. , Dawson D.V. , Perlmutter D.H. , Davis P.B. (1999) Chain length of the polylysine in receptor‐targeted gene transfer complexes affects duration of reporter gene expression both in vitro and in vivo. J Biol Chem;274:4908–4916. 9988733 10.1074/jbc.274.8.4908

[18] Naik R.J. , Chandra P. , Mann A. , Ganguli M. (2011) Exogenous and cell surface glycosaminoglycans alter DNA delivery efficiency of arginine and lysine homopeptides in distinctly different ways. J Biol Chem;286:18982–18993. 21471199 10.1074/jbc.M111.227793 PMC3099713

[19] Kwok A. , Eggimann G.A. , Reymond J.L. , Darbre T. , Hollfelder F. (2013) Peptide dendrimer/lipid hybrid systems are efficient DNA transfection reagents: structure–activity relationships highlight the role of charge distribution across dendrimer generations. ACS Nano;7:4668–4682. 23682947 10.1021/nn400343z PMC3715887

[20] Tagalakis A.D. , Kenny G.D. , Bienemann A.S. , McCarthy D. , Munye M.M. , Taylor H. , Wyatt M.J. , Lythgoe M.F. , White E.A. , Hart S.L. (2014) PEGylation improves the receptor‐mediated transfection efficiency of peptide‐targeted, self‐assembling, anionic nanocomplexes. J Control Release;174:177–187. 24269968 10.1016/j.jconrel.2013.11.014

[21] Tagalakis A.D. , McAnulty R.J. , Devaney J. , Bottoms S.E. , Wong J.B. , Elbs M. , Writer M.J. , Hailes H.C. , Tabor A.B. , O'Callaghan C. , Jaffe A. , Hart S.L. (2008) A receptor‐targeted nanocomplex vector system optimized for respiratory gene transfer. Mol Ther;16:907–915. 18388925 10.1038/mt.2008.38

[22] Shukla R.S. , Qin B. , Cheng K. (2014) Peptides used in the delivery of small noncoding RNA. Mol Pharm;11:3395–3408. 25157701 10.1021/mp500426r PMC4186677

[23] Hart S.L. , Arancibia‐Carcamo C.V. , Wolfert M.A. , Mailhos C. , O'Reilly N.J. , Ali R.R. , Coutelle C. , George A.J. , Harbottle R.P. , Knight A.M. , Larkin D.F. , Levinsky R.J. , Seymour L.W. , Thrasher A.J. , Kinnon C. (1998) Lipid‐mediated enhancement of transfection by a nonviral integrin‐targeting vector. Hum Gene Ther;9:575–585. 9525318 10.1089/hum.1998.9.4-575

[24] Tagalakis A.D. , Saraiva L. , McCarthy D. , Gustafsson K.T. , Hart S.L. (2013) Comparison of nanocomplexes with branched and linear peptides for siRNA delivery. Biomacromolecules;14:761–770. 23339543 10.1021/bm301842j

[25] Kenny G.D. , Villegas‐Llerena C. , Tagalakis A.D. , Campbell F. , Welser K. , Botta M. , Tabor A.B. , Hailes H.C. , Lythgoe M.F. , Hart S.L. (2012) Multifunctional receptor‐targeted nanocomplexes for magnetic resonance imaging and transfection of tumours. Biomaterials;33:7241–7250. 22809644 10.1016/j.biomaterials.2012.06.042

[26] Reynolds A. , Leake D. , Boese Q. , Scaringe S. , Marshall W.S. , Khvorova A. (2004) Rational siRNA design for RNA interference. Nat Biotechnol;22:326–330. 14758366 10.1038/nbt936

[27] Howard K.A. , Rahbek U.L. , Liu X. , Damgaard C.K. , Glud S.Z. , Andersen M.O. , Hovgaard M.B. , Schmitz A. , Nyengaard J.R. , Besenbacher F. , Kjems J. (2006) RNA interference in vitro and in vivo using a novel chitosan/siRNA nanoparticle system. Mol Ther;14:476–484. 16829204 10.1016/j.ymthe.2006.04.010

[28] Sundaram S. , Viriyayuthakorn S. , Roth C.M. (2005) Oligonucleotide structure influences the interactions between cationic polymers and oligonucleotides. Biomacromolecules;6:2961–2968. 16283715 10.1021/bm0502314 PMC2519154

[29] Hasumi H. , Akasaka K. , Hatano H. , Hiromi K. (1973) Use of 9‐aminoacridine as a probe in a kinetic study of DNA‐poly‐L‐lysine interaction. Biochem Biophys Res Commun;50:992–998. 4734820 10.1016/0006-291x(73)91504-0

[30] Tagalakis A.D. , He L. , Saraiva L. , Gustafsson K.T. , Hart S.L. (2011) Receptor‐targeted liposome‐peptide nanocomplexes for siRNA delivery. Biomaterials;32:6302–6315. 21624650 10.1016/j.biomaterials.2011.05.022

[31] Shim G. , Han S.E. , Yu Y.H. , Lee S. , Lee H.Y. , Kim K. , Kwon I.C. , Park T.G. , Kim Y.B. , Choi Y.S. , Kim C.W. , Oh Y.K. (2011) Trilysinoyl oleylamide‐based cationic liposomes for systemic co‐delivery of siRNA and an anticancer drug. J Control Release;155:60–66. 20971142 10.1016/j.jconrel.2010.10.017

[32] Tagalakis A.D. , Lee do H.D. , Bienemann A.S. , Zhou H. , Munye M.M. , Saraiva L. , McCarthy D. , Du Z. , Vink C.A. , Maeshima R. , White E.A. , Gustafsson K. , Hart S.L. (2014) Multifunctional, self‐assembling anionic peptide‐lipid nanocomplexes for targeted siRNA delivery. Biomaterials;35:8406–8415. 24985735 10.1016/j.biomaterials.2014.06.003

[33] Tagalakis A.D. , Castellaro S. , Zhou H. , Bienemann A. , Munye M.M. , McCarthy D. , White E.A. , Hart S.L. (2015) A method for concentrating lipid peptide DNA and siRNA nanocomplexes that retains their structure and transfection efficiency. Int J Nanomed;10:2673–2683. 10.2147/IJN.S78935 PMC4388080 25878500

[34] Mann A. , Richa R. , Ganguli M. (2008) DNA condensation by poly‐L‐lysine at the single molecule level: role of DNA concentration and polymer length. J Control Release;125:252–262. 18068848 10.1016/j.jconrel.2007.10.019

[35] Nayvelt I. , Thomas T. , Thomas T.J. (2007) Mechanistic differences in DNA nanoparticle formation in the presence of oligolysines and poly‐L‐lysine. Biomacromolecules;8:477–484. 17291071 10.1021/bm0605863 PMC2548297

[36] Korolev N. , Berezhnoy N.V. , Eom K.D. , Tam J.P. , Nordenskiold L. (2009) A universal description for the experimental behavior of salt‐(in)dependent oligocation‐induced DNA condensation. Nucleic Acids Res;37:7137–7150. 19773427 10.1093/nar/gkp683 PMC2790876

[37] Mann A. , Thakur G. , Shukla V. , Singh A.K. , Khanduri R. , Naik R. , Jiang Y. , Kalra N. , Dwarakanath B.S. , Langel U. , Ganguli M. (2011) Differences in DNA condensation and release by lysine and arginine homopeptides govern their DNA delivery efficiencies. Mol Pharm;8:1729–1741. 21780847 10.1021/mp2000814

[38] Wolfert M.A. , Seymour L.W. (1996) Atomic force microscopic analysis of the influence of the molecular weight of poly(L)lysine on the size of polyelectrolyte complexes formed with DNA. Gene Ther;3:269–273. 8646559

[39] Danielsen S. , Varum K.M. , Stokke B.T. (2004) Structural analysis of chitosan mediated DNA condensation by AFM: influence of chitosan molecular parameters. Biomacromolecules;5:928–936. 15132683 10.1021/bm034502r

[40] Golan R. , Pietrasanta L.I. , Hsieh W. , Hansma H.G. (1999) DNA toroids: stages in condensation. Biochemistry;38:14069–14076. 10529254 10.1021/bi990901o

[41] Bishop N.E. (1997) An Update on Non‐clathrin‐coated Endocytosis. Rev Med Virol;7:199–209. 10398484 10.1002/(sici)1099-1654(199712)7:4<199::aid-rmv203>3.0.co;2-f

[42] Jin Y. , Song Y. , Zhu X. , Zhou D. , Chen C. , Zhang Z. , Huang Y. (2012) Goblet cell‐targeting nanoparticles for oral insulin delivery and the influence of mucus on insulin transport. Biomaterials;33:1573–1582. 22093292 10.1016/j.biomaterials.2011.10.075

[43] Medina‐Kauwe L.K. , Xie J. , Hamm‐Alvarez S. (2005) Intracellular trafficking of nonviral vectors. Gene Ther;12:1734–1751. 16079885 10.1038/sj.gt.3302592

[44] Bolcato‐Bellemin A.L. , Bonnet M.E. , Creusat G. , Erbacher P. , Behr J.P. (2007) Sticky overhangs enhance siRNA‐mediated gene silencing. Proc Natl Acad Sci U S A;104:16050–16055. 17913877 10.1073/pnas.0707831104 PMC2042160

[45] Gratton S.E. , Ropp P.A. , Pohlhaus P.D. , Luft J.C. , Madden V.J. , Napier M.E. , DeSimone J.M. (2008) The effect of particle design on cellular internalization pathways. Proc Natl Acad Sci U S A;105:11613–11618. 18697944 10.1073/pnas.0801763105 PMC2575324

[46] Decuzzi P. , Ferrari M. (2008) The receptor‐mediated endocytosis of nonspherical particles. Biophys J;94:3790–3797. 18234813 10.1529/biophysj.107.120238 PMC2367199

[47] Adami R.C. , Collard W.T. , Gupta S.A. , Kwok K.Y. , Bonadio J. , Rice K.G. (1998) Stability of peptide‐condensed plasmid DNA formulations. J Pharm Sci;87:678–683. 9607943 10.1021/js9800477

[48] McKenzie D.L. , Smiley E. , Kwok K.Y. , Rice K.G. (2000) Low molecular weight disulfide cross‐linking peptides as nonviral gene delivery carriers. Bioconjug Chem;11:901–909. 11087340 10.1021/bc000056i

[49] Wadhwa M.S. , Collard W.T. , Adami R.C. , McKenzie D.L. , Rice K.G. (1997) Peptide‐mediated gene delivery: influence of peptide structure on gene expression. Bioconjug Chem;8:81–88. 9026040 10.1021/bc960079q

[50] Kebbekus P. , Draper D.E. , Hagerman P. (1995) Persistence length of RNA. Biochemistry;34:4354–4357. 7535562 10.1021/bi00013a026

[51] Hagerman P.J. (1997) Flexibility of RNA. Annu Rev Biophys Biomol Struct;26:139–156. 9241416 10.1146/annurev.biophys.26.1.139

[52] Brissault B. , Leborgne C. , Guis C. , Danos O. , Cheradame H. , Kichler A. (2006) Linear topology confers in vivo gene transfer activity to polyethylenimines. Bioconjug Chem;17:759–765. 16704215 10.1021/bc050287v

[53] Lote A.R. , Kolhatkar V.R. , Insley T. , Král P. , Kolhatkar R. (2014) Oligospermines and Nucleic Acid Interaction: A Structure Property Relationship Study. ACS Macro Letters;3:829–833. 10.1021/mz500358w 35590709

[54] Kadlecova Z. , Rajendra Y. , Matasci M. , Baldi L. , Hacker D.L. , Wurm F.M. , Klok H.A. (2013) DNA delivery with hyperbranched polylysine: a comparative study with linear and dendritic polylysine. J Control Release;169:276–288. 23379996 10.1016/j.jconrel.2013.01.019

[55] Patil M.L. , Zhang M. , Betigeri S. , Taratula O. , He H. , Minko T. (2008) Surface‐modified and internally cationic polyamidoamine dendrimers for efficient siRNA delivery. Bioconjug Chem;19:1396–1403. 18576676 10.1021/bc8000722

[56] Grayson A.C. , Doody A.M. , Putnam D. (2006) Biophysical and structural characterization of polyethylenimine‐mediated siRNA delivery in vitro. Pharm Res;23:1868–1876. 16845585 10.1007/s11095-006-9009-2

[57] Leng Q. , Scaria P. , Lu P. , Woodle M.C. , Mixson A.J. (2008) Systemic delivery of HK Raf‐1 siRNA polyplexes inhibits MDA‐MB‐435 xenografts. Cancer Gene Ther;15:485–495. 18483501 10.1038/cgt.2008.29 PMC5502125

[58] Leng Q. , Scaria P. , Zhu J. , Ambulos N. , Campbell P. , Mixson A.J. (2005) Highly branched HK peptides are effective carriers of siRNA. J Gene Med;7:977–986. 15772938 10.1002/jgm.748

[59] Chou S.T. , Leng Q. , Scaria P. , Woodle M. , Mixson A.J. (2011) Selective modification of HK peptides enhances siRNA silencing of tumor targets in vivo. Cancer Gene Ther;18:707–716. 21818135 10.1038/cgt.2011.40 PMC3177007

[60] Chou S.T. , Leng Q. , Scaria P. , Kahn J.D. , Tricoli L.J. , Woodle M. , Mixson A.J. (2013) Surface‐modified HK:siRNA nanoplexes with enhanced pharmacokinetics and tumor growth inhibition. Biomacromolecules;14:752–760. 23360232 10.1021/bm3018356 PMC3595641

[61] Chou S.T. , Hom K. , Zhang D. , Leng Q. , Tricoli L.J. , Hustedt J.M. , Lee A. , Shapiro M.J. , Seog J. , Kahn J.D. , Mixson A.J. (2014) Enhanced silencing and stabilization of siRNA polyplexes by histidine‐mediated hydrogen bonds. Biomaterials;35:846–855. 24161165 10.1016/j.biomaterials.2013.10.019 PMC3920840

[62] Katas H. , Alpar H.O. (2006) Development and characterisation of chitosan nanoparticles for siRNA delivery. J Control Release;115:216–225. 16959358 10.1016/j.jconrel.2006.07.021

