# Supplementary material for: Systematic Comparisons of Formulations of Linear Oligolysine Peptides with siRNA and Plasmid DNA
Source: Chem Biol Drug Des. 2016 Feb 1;87(5):747–63. doi: 10.1111/cbdd.12709 (PMC4991294; doi:10.1111/cbdd.12709)
Supplement: Supplementary file 1 — Figure S1. The binding of the linear lysine peptides to (A) pDNA and (B) siRNA at lower N/P ratios. Figure S2. The dissociation properties of linear lysine pDNA complexes. Figure S3. The dissociation properties of linear lysine siRNA complexes. Figure S4. siRNA transfection efficiency mediated by the linear lysine peptides with siRNA complexes. [file CBDD-87-747-s001.docx]

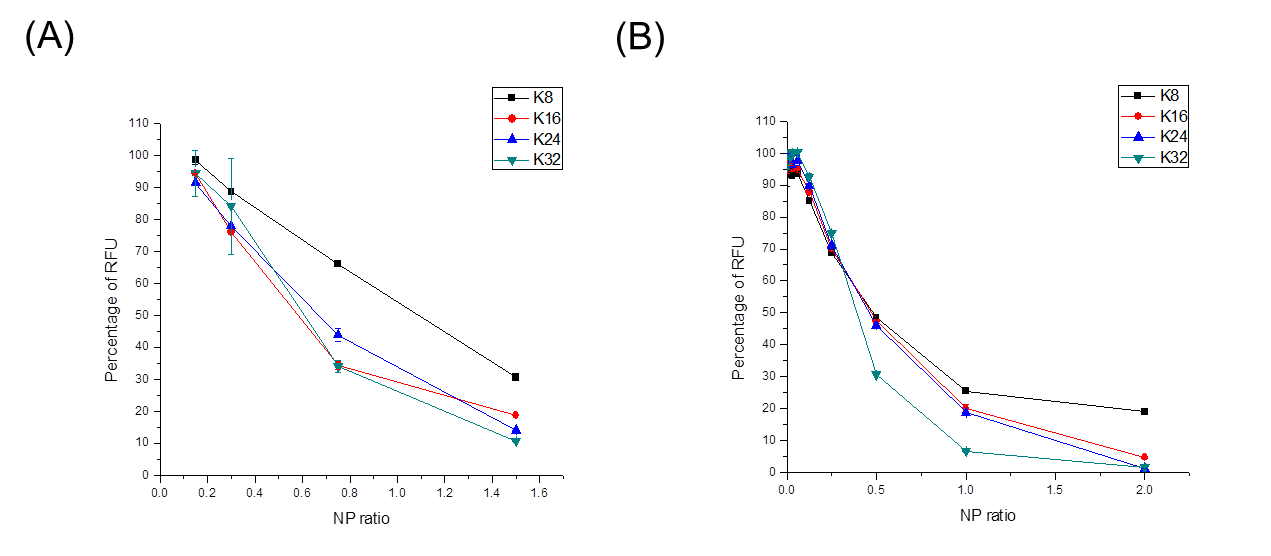


**Supplementary Figure 1. The binding of the linear lysine peptides to (A) pDNA and (B) siRNA at lower N/P ratios.** The linear lysine peptides were mixed with PicoGreen-labelled pDNA or siRNA at different N/P ratios for 30 minutes. The fluorescence intensity of the complexes were then measured and normalised with the naked pDNA or siRNA. The formulations of the complexes are expressed as an N/P ratio.


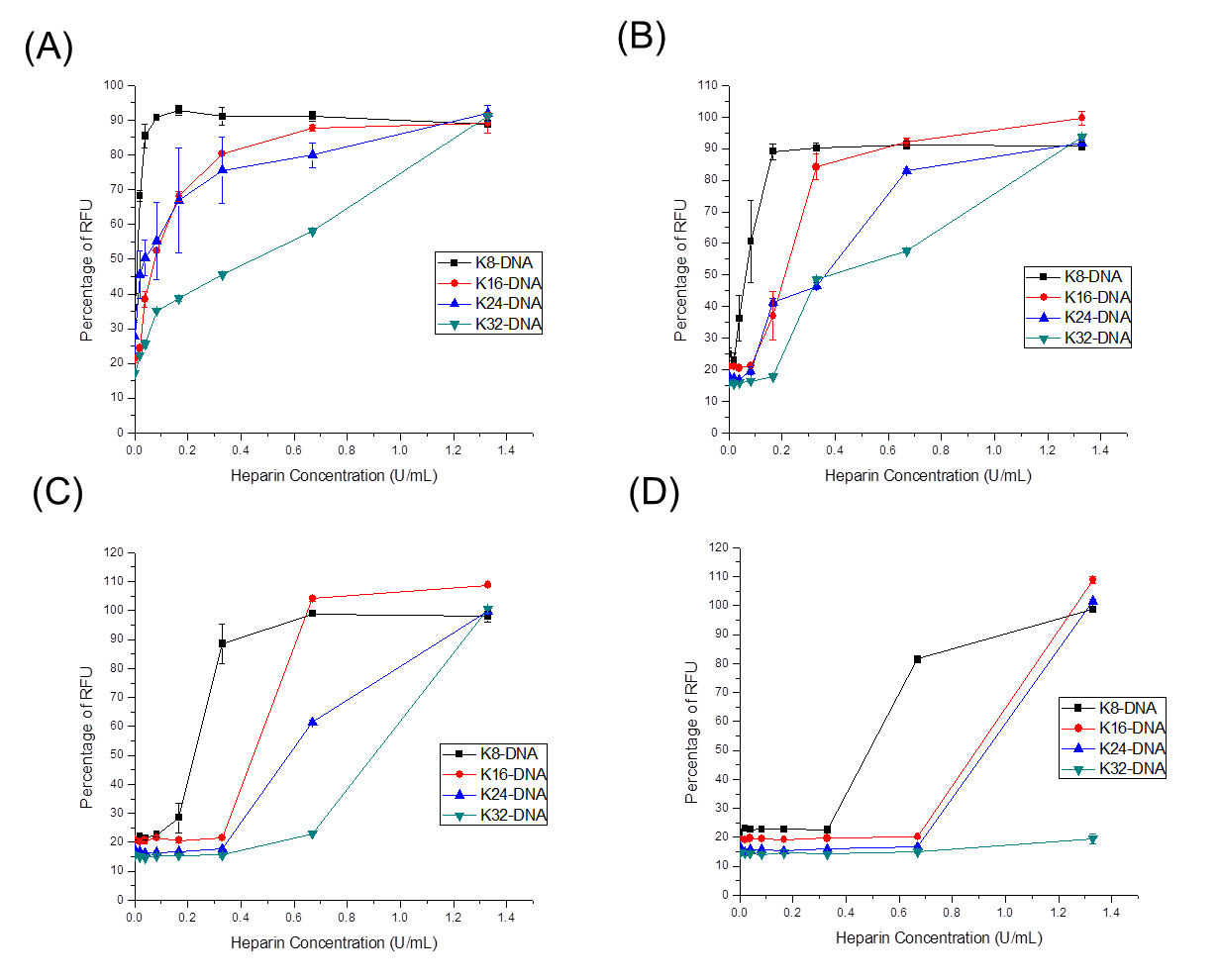


**Supplementary Figure 2. The dissociation properties of linear lysine pDNA complexes.** K8, K16, K24 and K32 peptides were mixed with PicoGreen-labelled pDNA at different N/P ratios for 30 minutes. Different concentrations of heparin were added to the complexes and the fluorescence intensity of the complexes was measured. **(A)** the lysine peptide pDNA complexes at an N/P ratio of 1.5, **(B)** the lysine peptide pDNA complexes at an N/P ratio of 3, **(**C) the lysine peptide pDNA complexes at an N/P ratio of 6 and **(**D) the lysine peptide pDNA complexes at an N/P ratio of 12.


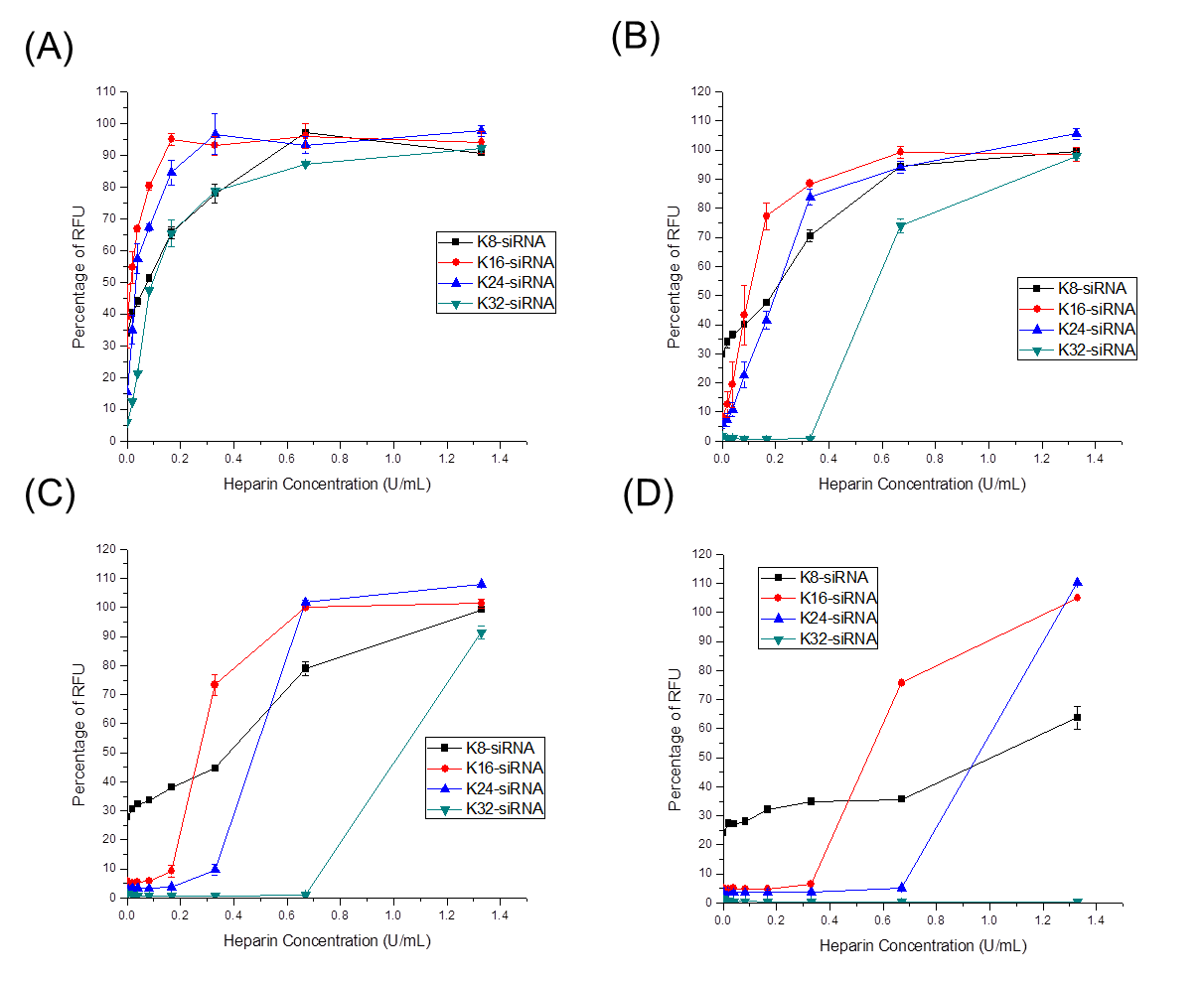


**Supplementary Figure 3. The dissociation properties of linear lysine siRNA complexes.** K8, K16, K24 and K32 peptides were mixed with PicoGreen-labelled siRNA at different N/P ratios for 30 minutes. Different concentrations of heparin were added to the complexes and the fluorescence intensity of the complexes was measured. **(A)** the lysine peptide siRNA complexes at an N/P ratio of 1, **(B)** the lysine peptide siRNA complexes at an N/P ratio of 2, **(C)** the lysine peptide siRNA complexes at an N/P ratio of 4 and **(D)** the lysine peptide siRNA complexes at an N/P ratio of 8.

**
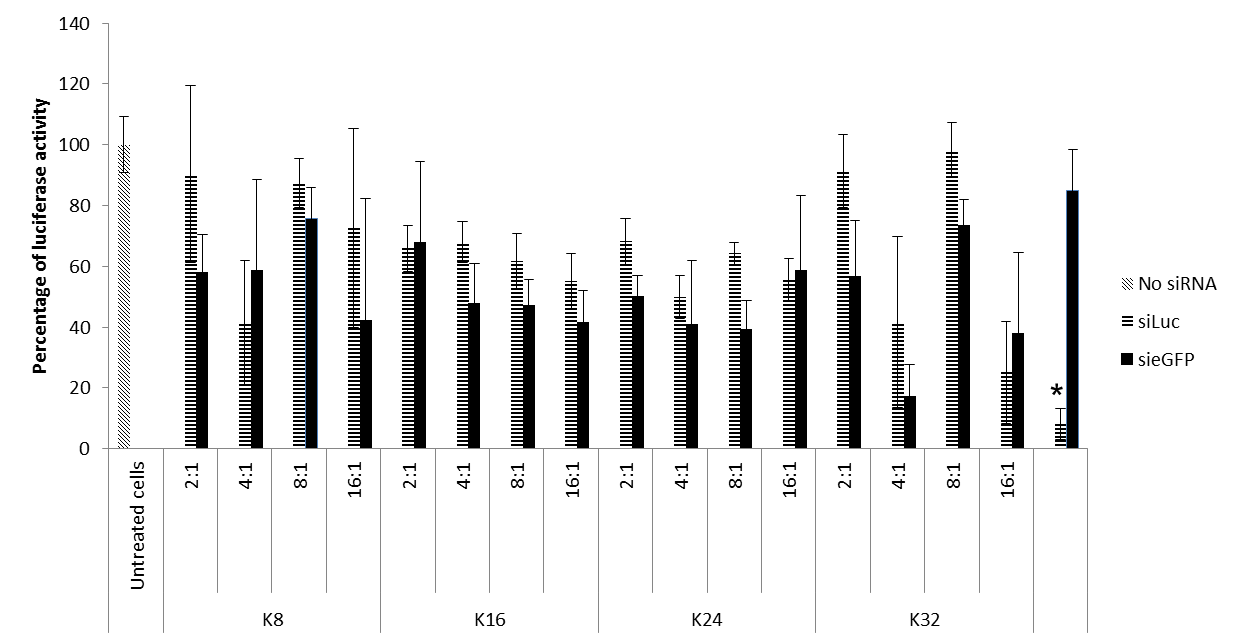
**

**Supplementary Figure 4. siRNA transfection efficiency mediated by the linear lysine peptides with siRNA complexes.** Neuro-2A luciferase expressing cells were seeded 24 hours before transfection. The complexes were made by mixing the linear lysine peptides (K8, K16, K24 and K32) with siRNA targeting luciferase (siLuc) at different N/P ratios for 30 minutes. Complexes with siRNA targeting eGFP (sieGFP) were used as a control to assess non-specific gene silencing mediated by the complexes. Following removal of full growth medium, complexes were overlaid to the cells for 4 hours. After removing the transfection complexes, full growth medium was added to the cells. Luciferase activity in the cells was analysed 24 hours post-transfection to estimate the transfection efficiencies of the complexes. The formulations of the complexes are expressed as an N/P ratio. * denotes the significant difference of the percentage of luciferase activity between the transfected cells with L2000 siLuc and L2000 sieGFP complexes (p<0.05).
